# Gender and age group modified association of dental health indicators with total occlusal force among Korean elders

**DOI:** 10.1186/s12903-021-01928-y

**Published:** 2021-11-08

**Authors:** Christine Hyun Jin Lee, Huong Vu, Hyun-Duck Kim

**Affiliations:** 1 Department of Preventive and Social Dentistry, College of Dentistry, Seoul National University, 28 Younkeon‐Dong, Chongro‐Ku, Seoul, 03080 Korea; 2 Dental Research Institute, College of Dentistry, Seoul National University, Seoul, Korea

**Keywords:** Occlusal force, Dental health, Natural teeth, Rehabilitated teeth, Elder

## Abstract

**Background:**

This study aimed to investigate the distribution of objective total occlusal force (TOF) and its association with dental health indicators: dental status, number of natural teeth (NT), natural and rehabilitated teeth, natural posterior teeth (NT-Post), and natural and rehabilitated posterior teeth among Korean elders after controlling for various confounders encompassing socio-demographic factors, behavioral factors and health/oral health factors.

**Methods:**

This cross-sectional study recruited 551 elders from the Sungbook-Gu health education cohort. TOF was measured using Prescale II as an outcome variable. Dental health indicators assessed by dentists were the main explanatory variables. Analysis of covariance and multivariable linear regression models were applied to evaluate the adjusted association of dental health indicators with TOF. Gender and age group stratified analyses were also applied.

**Result:**

TOF was higher in dentate elders than denture wearers in males and younger elders. The adjusted mean of TOF and standard error was 464.24 ± 17.15 N for dentate elders, 297.15 ± 28.85 N for partial denture wearers, 280.42 ± 47.71 N for complete denture wearers. Among all dental health indicators, NT-Post showed the highest association with TOF (partial r = 0.330, *p* < 0.001, R^2^ = 0.15), followed by NT (partial r = 0.329, *p* < 0.001, R^2^ = 0.16). Older elders highlighted the association of NT (partial r = 0.37, *p* < 0.001, R^2^ = 0.18). Males decreased the association of NT (partial r = 0.30, *p* < 0.001, R^2^ = 0.20) and NT-Post (partial r = 0.29, *p* < 0.001, R^2^ = 0.20).

**Conclusion:**

TOF was significantly associated with dental health indicators, and its association was modified by sex and age group.

## Background

One of the global key goals of oral health 2020 by the FDI is to reduce the number of individuals experiencing dental functional disorders such as chewing, swallowing, and speaking [1]. According to the reports from the ‘Global burden of oral conditions in 1990–2010’, oral conditions were ranked in the top 100 causes of disability-adjusted life years because of population growth and aging: severe tooth loss due to carious or periodontal conditions ranked 36th [2].

Since 2000, South Korea has become an aging society and is expected to become a super-aged society in the future [3]. In 2016, amongst the edentulous Korean elderly, 87.8% were denture wearers, and 12.2% were non-denture wearers [3]. New challenges are arising for elders’ dental health including maintenance of their natural teeth and rehabilitation of missing teeth [4].

Statistics showed that Koreans aged over 65 wearing a mandibular complete denture experienced oral problems such as chewing and pronouncing: the prevalence of oral problems ranged up to 30–40% [5]. The Korean National Health Insurance has covered two implants and dentures for Koreans aged over 65. Although rehabilitation such as implants and dentures would have improved in their oral function, the proportion of Korean elders who had limitations in chewing ability was still at 45.8% [6]. Low masticatory ability due to tooth loss had a negative effect on nutritional status [7]. Rehabilitation of lost teeth could reduce the risk of cognitive impairment because it could potentially increase the occlusal force [8]. These statistics outline that the dental problems could be related to occlusal force, which indicated that total occlusal force is important.

Many epidemiological studies used dental health indicators (DHI) such as dental status (DS), natural teeth (NT), and natural and rehabilitated teeth (NRT) as a surrogate of the objective occlusal force [9, 10]. Some Japanese studies reported the association of objective occlusal force with bilaterally missing molars [11], clenching [12], dentate status [13]. However, the association of DHI including DS, TN, NRT with total occlusal force has not been reported. Moreover, the influence of age on TOF has not been clarified yet. One study showed no or little relation of occlusal force across age groups [14], another study showed decreased occlusal force by aging [15]. Japanese evidence of higher impact on TOF in males than in females [13, 16] should need more similar evidence in other countries to establish the candid evidence, according to the consistency criteria of Sir Bradford Hill [17]. Thus, the association of DHI including DS, TN, NRT with TOF should be evaluated, focusing on the effect modification of its association by gender and age group. Hence, this study aims to evaluate and compare the association of TOF with DHI, including DS, TN and NRT and investigate the modified association by gender and age group among Korean elders after controlling for various confounders including age, gender, education, smoking, alcohol drinking, and metabolic syndrome.

## Methods

### Ethical considerations and study design

This cross-sectional study was approved by the Institutional Review Board for Human Subjects at the Seoul National University School of Dentistry (Approval Number: S-020190017) and the Seoul National University Hospital Biomedical Research Institute (IRB Approval No., C-1803-117-932). All participants provided written informed consent of their own accord. This study was the baseline (2018–2019) of the community health education cohort [18]. Participants were recruited from the residents in Sungbook-Gu after advertisement for several weeks in advance of the survey. The survey was conducted at a community health center in Sungbook-Gu, Seoul, which was a combined Medical and Dental health promotion research. Systemic health status and oral health status were assessed by trained medical and dental health professionals who received calibration training beforehand.

### Participants

Participants were recruited from 10 sub-districts in Sungbook-Gu, which were selected by cluster sampling methods. The inclusion criteria were six-fold: (1) aged 65 and above who lived in Sungbook-Gu, (2) elders without critical diseases such as cancer and paralysis, (3) elders without any problem for communication, (4) elders with a willingness to follow the recommendation of the cohort procedures, (5) elders with all of the information considered in this study. Exclusion criteria were (1) edentulous people without dentures, (2) elders with muscular disorders or joint problems which can affect muscle of mastication and indirectly affect TOF. A total of 743 elders in Sungbook-Gu were recruited for this cohort. Out of them, 551 elders met the inclusion criteria of this study.

### Assessment of total occlusal force

According to the manufacturer’s guideline, TOF was assessed by two dentists using a commercial occlusal film kit (Dental Prescale II, GC Corporation, Tokyo, Japan). This horseshoe shape film (pressure-sensitive sheet) consists of two polyethylene terephthalate films with many pigment-containing microcapsules between them. When the participant bit, the microcapsules were broken and released the red color. According to the pressure applied, the intensity of the color was different [19].

The film was selected from medium or large size predetermined by positioning the film inside the patient’s mouth, and the patient’s neck was unrestrained but supported by the examiner's nonworking hand for upright positioning. Then, the film was placed into the participant’s mouth so that the midline was coincide with the midline of the arch and encompassed the entire dentition.

The participants were asked to bite on both sides with maximal intercuspation onto the film with maximum force for 3 s according to the guidelines for the kit. Then, the film was kept in a light-resistant box until measurement on the same day. For denture wearers, measurement was performed while dentures were in the mouth.

**Fig. 1:**
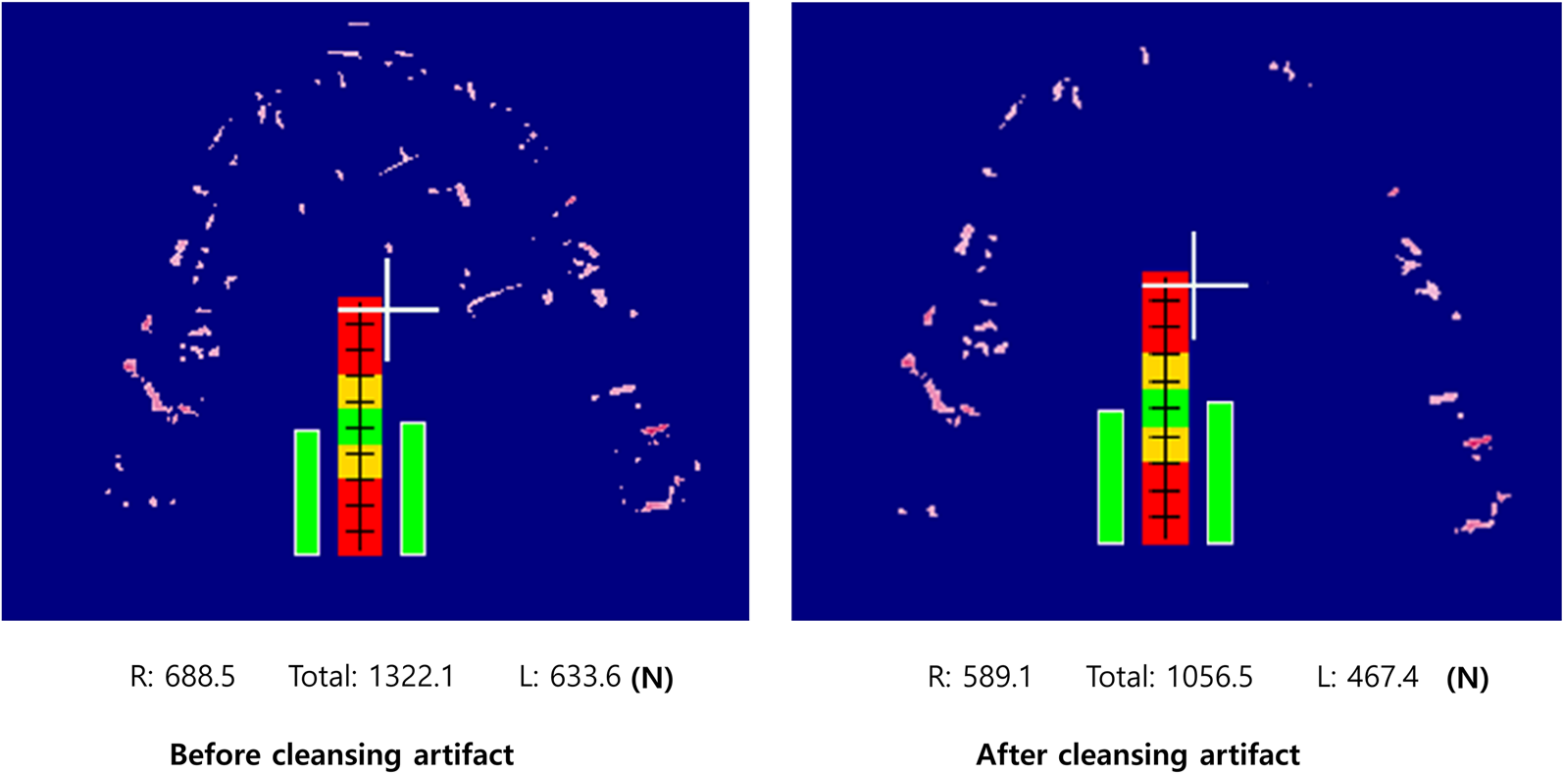
Occlusal contact status and occlusal force (Newton) in bite films before and after cleansing artifacts. Artifacts placed out of occlusal area showed white or slightly red colors. Only red colored spots in occlusal area remained as genuine occlusal spots. Dark red colored spot showed the highest force, followed by red spots

Afterward, the film was scanned using an optical image scanner (GT-X830, Epson Corp.) into Bite Force Analyzer software, which was provided by the manufacturer for measurement. During clenching with the upper and lower teeth, the sheet can be bent or slid, causing color development in parts that are not actually in occlusal contact. Therefore, these noises (artifacts) were assessed and cleansed by well-trained dentists. (Fig. 1) [20]. After cleansing artifacts, TOF was reduced by 21–26% (n = 10). The clear red spots with the shape of occlusal traces were determined as genuine occlusal contact. Then, the measurement of TOF was done automatically by the program. The TOF was evaluated in Newton (N).

For reliability of TOF testing, 10% of the films were planned to be re-test. Intra-examiner reliability (ICC) was 0.967 (n = 50) and inter-examiner reliability was 0.956 (n = 20).

### Assessment of dental health indicators

The DS, NT, NRT were selected as DHI. DS was classified into three groups: the dentate elders, removable partial denture (PD) wearers, and complete denture (CD) wearers. PD denotes having any removable PD without a complete denture. CD denotes wearing at least one CD. During the oral examination, dentists counted NT and NRT using dental explorer and naked eye under the blue light in the mobile dental unit chair. Pontic of fixed bridge and implants (artificial teeth) were considered as rehabilitated teeth. NRT for posterior teeth including premolar and molar (NRT-Post) and NT for posterior teeth including premolar and molar (NT-Post) were considered as the teeth number in premolars and molars. NRT and NT were categorized into three groups: low group with (0–15 teeth), moderate group with (16–23 teeth), high group with (24–32 teeth). Although a report insisted that third molars were usually not functional to occlusal force [21], 23.5% of all participants (n = 135) had third molars and 26.7% of them (n = 36) were functional. Hence, third molars were included in this study.

According to the short dental arch concept, the minimum NRT number was set at 24 teeth or more for high NT and NTR group [22]. According to the modification of Eichner Index B3 [23], low NT and NRT groups were set at 0–15 teeth.

### Potential confounders

Socio-demographic factors such as age, gender, education, behavioral factors such as smoking and drinking and, medical and dental factors such as periodontitis and metabolic syndrome were potential confounders that were assessed by trained dentists and physicians. Age, gender, education level, smoking, and drinking were obtained from the interview in the survey. Metabolic syndrome defined by APT III guidelines was assessed through data measured from the medical examination and laboratory blood tests. Assessment of metabolic syndrome was having three or more components based on the following five components: obesity (body mass index (body kg/ height m2) ≥ 25) [24]; hypertriglyceridemia (triglycerides > 150 mg/dL); low HDL cholesterol (< 40 mg/dL for men and < 50 mg/dL for women) or on medication for dyslipidemia; high blood pressure (systolic: > 130 mmHg or diastolic: > 85 mmHg or on hypertension medication); and glycated hemoglobin HbA_1_C ≥ 5.3% or medication for diabetes [25].

Periodontitis was assessed using the clinical attachment loss (CAL) of individual tooth and tooth loss due to periodontitis according to the American Academy of Periodontology-European Federation of Periodontology (AAP-EFP) periodontal classification guideline using Staging and Grading of periodontitis [26]. CAL was calculated in consideration of both recession and pocket depth by the UNC probe measurement. Two sites per tooth (mesial and distal surfaces) were measured except for the second molars, which used only the mesial surface. The criteria for periodontitis were followed using the Staging and grading of periodontitis: Framework and proposal of a new classification and case definition [26]. Periodontitis was classified into two groups: No (Stages 1–2) and Yes (Stages 3–4). However, complexity factors such as using the radiographic bone loss were not considered in the classification of periodontitis.

### Statistical methods and analysis

Distribution in characteristics of the participants according to dental status was expressed using frequencies and percentages for categorical variables and mean values with standard deviations for continuous variables. The differences were evaluated using the chi-square test for categorical variables. T-test by two groups and analysis of variance (ANOVA) by three or more groups, including Bonferroni’s post hoc multiple comparison test, was applied for continuous variables. The outcome variable was TOF, and the main explanatory variables were DHI such as DS, NT, NRT, NT-Post, and NRT-Post. Although TOF was not normally distributed (Kolmogorov–Smirnov test, *p* < 0.001), a large number of participants (n = 551) made the parametric test available [27]. Correlation analysis was applied for the evaluation of relationships between DHI and TOF. This study performed a separate independent analysis to evaluate the association of each DHI such as DS, NT, NRT, NT-Post, and NRT-Post with TOF. Analysis of covariance (ANCOVA) was applied for evaluating the differences in adjusted values of TOF after controlling for various confounders encompassing socio-demographic factors such as education, age, gender, behavioral factors such as alcohol intake and smoking status, and health factors such as metabolic syndrome. Multivariable linear regression analysis was applied to evaluate the impact of association (partial-r) between TOF and DHI after controlling for various confounders. Stratified analyses were also applied for gender (males versus females) and age group (less than 75 years versus 75 years or older). The interaction effect between NRT and DS on TOF was also evaluated. In high NRT (24–32) group, DS was three groups: dentate (n = 285), PD (n = 112) and CD (n = 46). In the moderate NRT (16–23) group, PD (n = 18) and CD (n = 3) merged into the Denture group (n = 21), because the number of CD was too small. Since the total number of low NRT (< 16) group was 17, this group was not considered in the analysis. Statistical significance was set at *p* value < 0.05. Statistical software used was IBM SPSS version 26.

## Results

### Characteristics of the participants according to dental status

Total 551 participants (165 males and 386 females) had a mean age of 75.8 with a standard deviation (SD) of 5.2 years (Table 1). The dentate group, compared to the denture group, was younger and less smoking, less periodontitis and had more metabolic syndrome, higher NT and NT-Post, but less NRT and NRT-Post (*p* < 0.05) (Table 1). The dentate group was more educated, drinking less, than the dentures, but the difference was not statistically significant (*p* > 0.05).

**Table 1:** Characteristics of participants according to dental status (N = 551)

	Dentate (n = 371)	Partial denture (n = 131)	Complete denture (n = 49)	*p* value
NT*	**22.45 ± 5.68**^**a**^	**13.67 ± 5.35**^**b**^	**5.75 ± 3.10**^**c**^	**< 0.001**
NRT*	**25.38 ± 4.50**^**a**^	**26.66 ± 2.82**^**b**^	**27.16 ± 1.92**^**b**^	**< 0.001**
NT-Post*	**11.86 ± 4.29**^**a**^	**5.95 ± 3.17**^**b**^	**2.45 ± 1.86**^**c**^	**< 0.001**
NRT-Post*	**13.78 ± 3.67**^**a**^	**15.00 ± 2.24**^**b**^	**15.31 ± 1.52**^**b**^	**< 0.001**
Age*, year	**75.39 ± 5.08**	**76.40 ± 5.15**	**77.36 ± 6.24**	**0.016**
Sex				0.187
Male	102 (61.8)	45 (27.3)	18 (10.9)	
Female	269 (69.7)	86 (22.3)	31 (8.0)	
Education level				0.135
High school or more	98 (74.2)	26 (19.7)	8 (6.1)	
Junior school or less	273(65.2)	105 (25.1)	41 (9.8)	
Smoking**				**0.008**
No	**278 (71.1)**	**85 (21.7)**	**28 (7.2)**	
Yes	**93 (58.1)**	**46 (28.7)**	**21 (13.1)**	
Alcohol drinking^†^				0.140
No	122 (64.9)	43 (2.9)	23 (12.2)	
Yes	249(68.6)	88(24.2)	26(7.2)	
Periodontitis^♦^				**< 0.001**
No	**113 (86.3)**	**14 (10.7)**	**4 (3.1)**	
Yes	**258 (61.4)**	**117 (27.9)**	**45 (10.7)**	
Metabolic syndrome^‡^				**0.004**
No	**144 (61.3)**	**60(25.5)**	**31(13.2)**	
Yes	**227 (71.8)**	**71(22.5)**	**18 (5.7)**	

Data are presented as numbers (raw percentage) for categorical variables and mean ± standard deviation for continuous variables*

Superscript^abc^ denotes same subgroup by Bonferroni’s post hoc multiple comparisons test in ANOVA

*p* value: obtained from chi-square test for categorical variables and ANOVA including Bonferroni post hoc multiple comparison test for continuous variables

**Bold** denotes statistically significant

*NT* number of natural teeth, *NRT* number of natural and rehabilitated teeth, *NT-Post* number of natural posterior teeth, *NRT-Post* number of natural and rehabilitated posterior teeth

**Smoking: “No” refers to never smoked, “Yes” refers to past and current smoker

^†^Alcohol drinking: “No” refers to drunken, “Yes” refers to past and current drinker

^♦^Periodontitis: followed by guideline “Staging and grading of periodontitis: Framework and proposal of a new classification and case definition” [23] classified into two groups: No (healthy or stage I–II) and Yes (stage III–IV)

^‡^ Metabolic syndrome: “No” refers to two or fewer factors, “Yes” refers to three or more factors among five factors: Obesity (body mass index (body kg/ height m^2^) ≥ 25); Total triglyceride ≥ 150 mg/dL; HDL cholesterol: Male < 40 mg/dL, Female < 50 mg/dL or medication for dyslipidemia; Hypertension: systolic blood pressure ≥ 130 mmHg or diastolic blood pressure ≥ 85 mmHg or medication for hypertension; Glycated hemoglobin ≥ 5.3% or medication for diabetes

### Crude and adjusted occlusal force according to dental health indicators

**Fig. 2:**
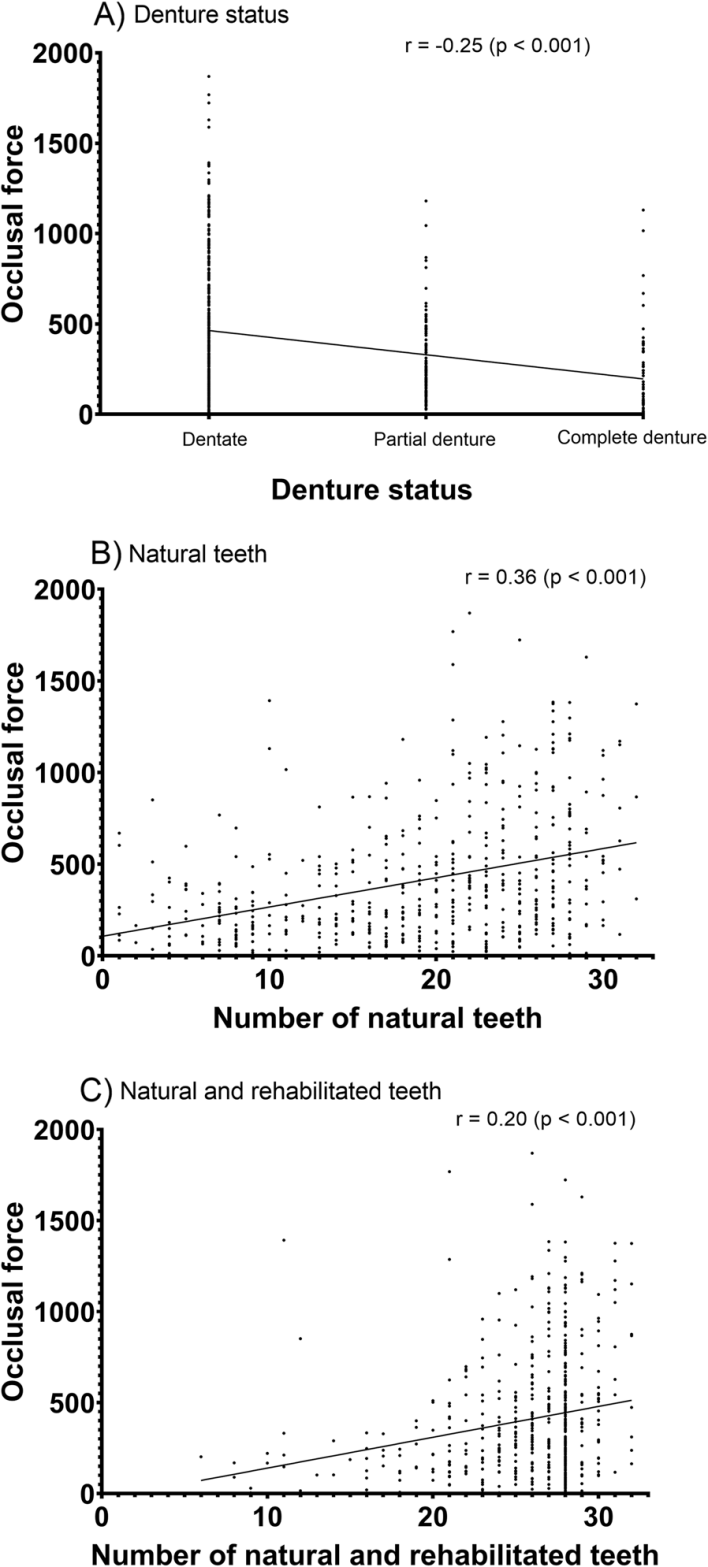
Total occlusal force according to dental status, natural teeth, natural and rehabilitated teeth (n = 551). Values were obtained from scatterplots to show the association between occlusal force and **A** denture status, **B** number of natural teeth, **C** number of natural and rehabilitated teeth. Pearson’s correlation coefficient (r) was obtained from correlation analysis

TOF was highly correlated with dental status (0: dentate; 1: PD; 2: CD), NT and NRT (Fig. 2): correlation coefficient (r) of − 0.25 for dental status, 0.36 for NT, and 0.20 for NRT.

According to Bonferroni’s post hoc multiple comparisons test in ANOVA, the crude and adjusted TOF were higher in dentate elders than in denture elders (*p* < 0.001) (Table 2). The crude mean of TOF with SD was 471.82 ± 368.00 N for dentate elders, 284.64 ± 217.36 N for PD, and 256.48 ± 246.31 N for CD (ANOVA, *p* < 0.001). The adjusted mean of TOF with standard error (SE) was 464.24 ± 17.15 N for dentate, 297.15 ± 28.85 N for PD, and 280.42 ± 47.71 N for CD (ANCOVA, *p* < 0.001). High NT and NRT showed significantly higher TOF as compared to moderate and low NT and NRT groups (*p* < 0.001). The adjusted mean and SE of TOF was 542.71 ± 29.09 N for high NT group, followed by 405.02 ± 22.84 N for moderate NT group and 262.87 ± 25.78 N for low NT group (ANCOVA, *p* < 0.001). Similarly, as to NRT group, the adjusted mean and SE of TOF was highest in the high NRT group at 431.09 ± 15.75 N, followed by the moderate NRT group at 311.30 ± 36.18 N and lower NRT group at 301.17 ± 81.49 N (ANCOVA, *p* = 0.005).

**Table 2:** Total occlusal force according to dental health indicators (N = 551)

Variable	N	Occlusal force (Newton)	
		Crude, mean ± SD	Adjusted, mean ± SE
Dental status			
Dentate	371	471.82 ± 368.00^a^	464.24 ± 17.15^a^
Partial denture	131	284.64 ± 217.36^b^	297.15 ± 28.85^b^
Complete denture	49	256.48 ± 246.31^b^	280.42 ± 47.71^b^
***p* value**		< 0.001	< 0.001
NT			
Low (0–15 teeth)	167	262.83 ± 218.83^a^	262.87 ± 25.78^a^
Moderate (16–23 teeth)	199	398.65 ± 333.28^b^	405.02 ± 22.84^b^
High (24–32 teeth)	185	550.24 ± 379.74^c^	542.71 ± 25.09^c^
***p* value**		< 0.001	< 0.001
NRT			
Low (0–15 teeth)	17	265.42 ± 347.83^a^	301.17 ± 81.49^a^
Moderate (16–23 teeth)	87	303.85 ± 282.99^a,b^	311.30 ± 36.18^a,b^
High (24–32 teeth)	447	433.90 ± 340.56^a,c^	431.09 ± 15.75^a,c^
***p* value**		< 0.001	0.005

*SD* standard deviation, *SE* standard error, *NT* number of natural teeth, *NRT* number of natural and rehabilitated teeth

*P* value obtained from ANOVA for crude value and ANCOVA for adjusted value controlling for age, sex, education, smoking, alcohol, periodontitis, metabolic syndrome

Superscript^abc^ denote same subgroup by Bonferroni’s post hoc multiple comparisons test

### Total occlusal force according to gender and age group

**Fig. 3:**
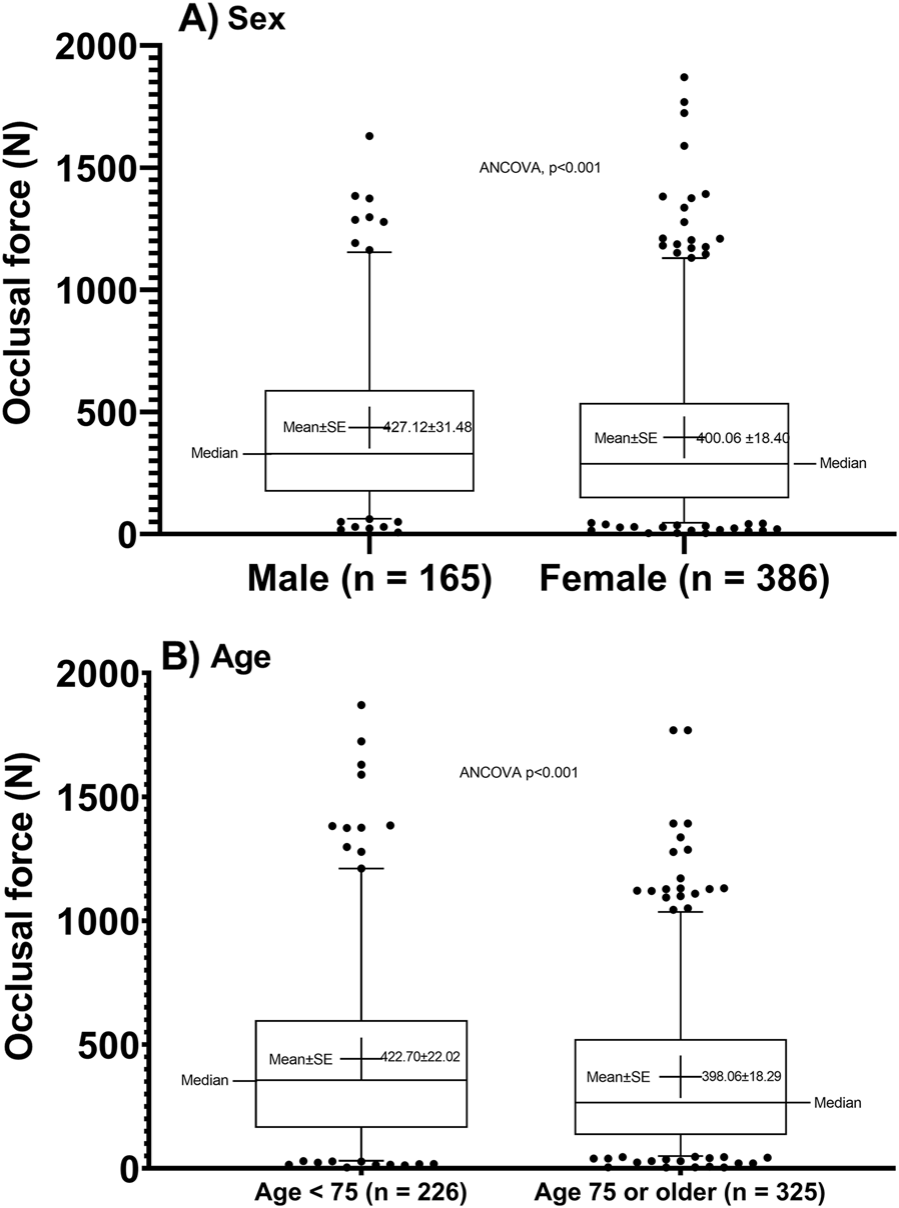
Occlusal force according to gender and age group. Box and whisker plot for stratified data for **A** gender (male versus female) and **B** age group (less than 75 years versus 75 years or older). Box denotes median, interquartile range values and whisker denotes 5–95% data points. Adjusted mean values (cross in the box plot) and standard error were obtained from ANCOVA

Males showed a higher adjusted mean and SE of TOF than females (ANCOVA, *p* < 0.001): 427.12 ± 31.48 N for males and 400.06 ± 18.40 N for females (Fig. 3). Younger elders aged less than 75 years also showed higher adjusted mean and SE of TOF than older elders aged 75 years or more (ANCOVA, *p* < 0.001): 422.70 ± 22.02 N for youngers and for 398.06 ± 18.29 N for older elders.

### Total occlusal force according to interaction between NRT group and dental status

**Fig. 4:**
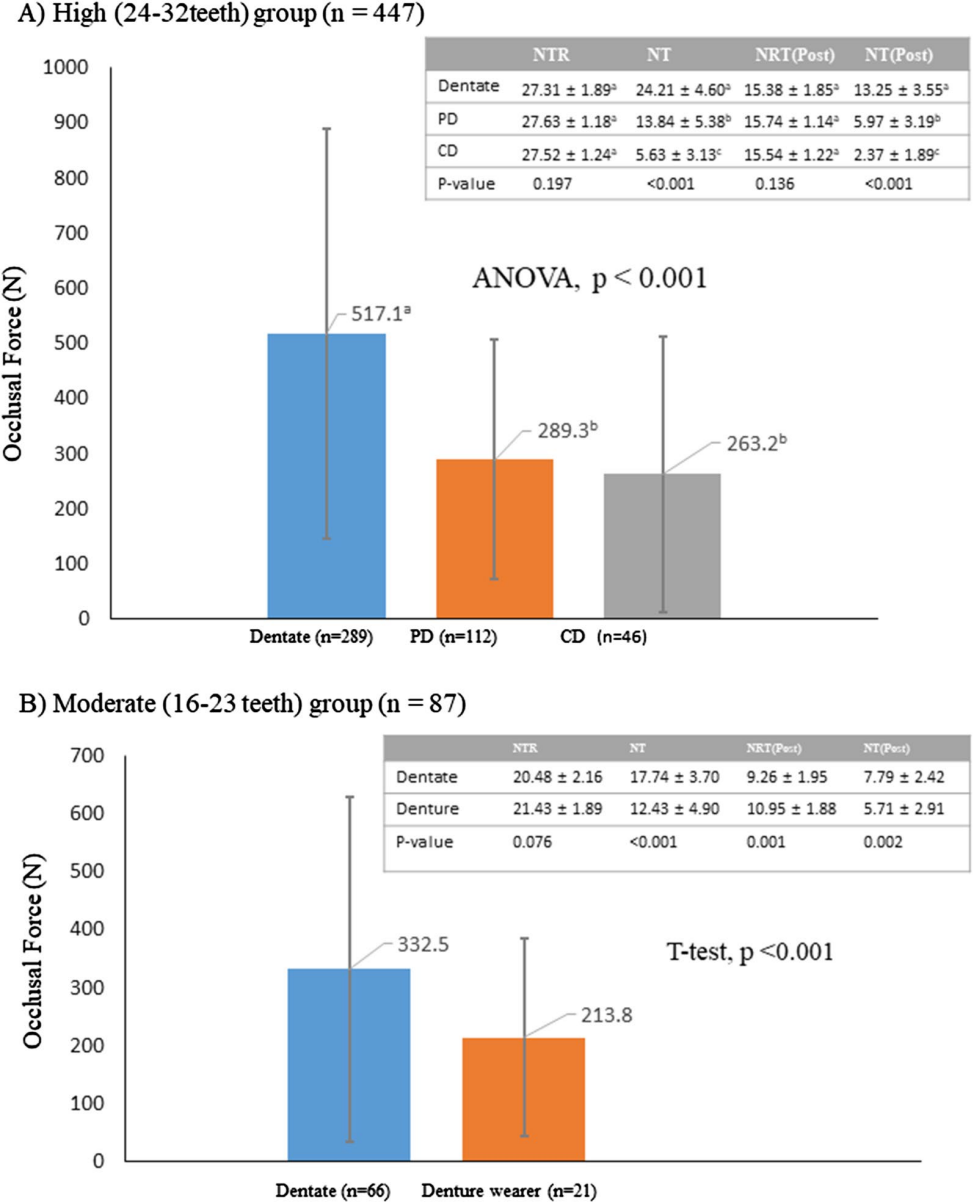
Occlusal force according to dental status with NRT (high and moderate groups). Two separate graphs to compared occlusal force with dental status in **A** high NRT group (24–32 teeth) and **B** moderate NRT group (16–23 teeth). A bar denotes mean value and whisker denotes standard deviation (SD). Superscript^abc^ denotes same subgroup by Bonferroni’s post hoc multiple comparisons test in ANOVA. Side table for high NRT group: ANOVA was applied to compare mean with SD of NT, NRT, NT-Post and NRT-Post across dental status. Side table for moderate NRT group: T-test was applied to compare mean with SD of NRT, NT, NT-Post and NRT-Post between dentate and denture (partial and complete denture) group

NRT group separate analysis showed that the dentate group showed higher TOF than the denture group in both high and moderate NRT groups (*p* < 0.05) (Fig. 4). In the high NRT group, higher TOF in the dentate group compare to the denture group was related to higher NT and NT-Post: NT was 24.21 for dentate, 13.84 for PD and 5.63 for CD, and NT-Post was 13.25 for dentate, 5.97 for PD and 2.37 for CD (ANOVA, *p* < 0.001). In the moderate NRT group, TOF was higher in dentate than in the denture group (T-test, *p* < 0.001). Although NTR was not statistically different between both groups (T-test, *p* = 0.076), NT was 17.74 for dentate and 12.43 for the denture, and NT-Post was 7.79 for dentate, 5.71 for denture (T-test, *p* = 0.002). Moreover, NRT and NRT-Post were neither related to TOF: NRT and NRT-Post were a bit higher in the denture group, but TOF was lower in denture group.

### Association of dental health indicators on total occlusal force

In total participants, the impact of NT-post was highest among all DHI (partial r = 0.330, *p* < 0.001), followed by NT (partial r = 0.329, *p* < 0.001), DS (partial r = − 0.217, *p* < 0.001) and NRT (partial r = 0.174, *p* < 0.001) & NRT-post (partial r = 0.176, *p* < 0.001) (Table 3).

**Table 3:** Comparison between association impact of dental health indicators on total occlusal force (n = 551)

Variable (range)	β	SE	Partial r	*p* value	R^2^
*Model 1*					
Dental status (0–2)	−** 116.44**	**22.54**	−** 0.22**	**< 0.001**	0.10
Age	− 3.89	2.74	− 0.06	0.14	
Sex	− 27.06	40.33	− 0.03	0.68	
Periodontitis	− 97.81	34.08	− 0.12	0.06	
Education	35.04	34.13	0.04	0.24	
Smoking	45.21	41.15	0.05	0.34	
Alcohol	21.54	30.54	0.07	0.43	
Metabolic syndrome	25.12	28.56	0.06	0.37	
*Model 2*					
NT (0–32)	**15.65**	**1.93**	**0.33**	**< 0.001**	0.16
Age	− 3.42	2.64	− 0.06	0.20	
Sex	− 43.51	39.03	− 0.05	0.27	
Periodontitis	− 42.30	34.16	− 0.05	0.22	
Education	8.87	33.28	0.01	0.79	
Smoking	63.19	39.87	0.07	0.11	
Alcohol	8.34	29.62	0.01	0.78	
Metabolic syndrome	33.62	27.44	0.05	0.22	
*Model 3*					
NRT(0–32)	**14.98**	**3.63**	**0.17**	**< 0.001**	0.08
Age	− 5.16	2.75	− 0.08	0.06	
Sex	− 36.34	40.69	− 0.04	0.37	
Periodontitis	−** 98.87**	**34.61**	−** 0.12**	**0.004**	
Education	38.59	34.42	0.05	0.26	
Smoking	36.25	41.40	0.04	0.38	
Alcohol	24.26	30.80	0.03	0.43	
Metabolic syndrome	49.93	28.63	0.08	0.08	
*Model 4*					
NT-Post (0–20)	**23.24**	**2.86**	**0.33**	**< 0.001**	0.15
Age	− 3.09	2.65	− 0.05	0.24	
Sex	− 32.46	38.99	− 0.04	0.41	
Periodontitis	− 44.94	34.04	− 0.06	0.19	
Education	19.06	33.11	0.03	0.57	
Smoking	54.69	39.73	0.06	0.17	
Alcohol	11.63	29.57	0.02	0.69	
Metabolic syndrome	35.52	27.43	0.06	0.20	
*Model 5*					
NRT-Post (0–20)	**18.36**	**4.42**	**0.176**	**< 0.001**	0.05
Age	− 5.22	2.74	− 0.08	0.06	
Sex	− 35.08	40.68	− 0.04	0.39	
Periodontitis	−** 98.16**	**34.63**	−** 0.12**	**0.005**	
Education	40.76	34.37	0.05	0.24	
Smoking	31.22	41.28	0.03	0.45	
Alcohol	25.48	30.77	0.04	0.41	
Metabolic Syndrome	50.10	28.63	0.08	0.08	

*NT* number of natural teeth, *NRT* number of natural and rehabilitated teeth, *NT-Post* number of natural posterior teeth, *NRT-Post* number of natural and rehabilitated posterior teeth, ***β*** regression coefficient; *SE* standard error for **β**

**Bold** denotes statistically significant

Partial r obtained from linear regression model for occlusal force adjusted for age, gender, smoking, alcohol, education, periodontitis and metabolic syndrome

Dental Status: 0 = Dentate; 1 = Partial denture; 2 = Complete denture

Model 1 for Dental status (0–2); Model 2 for NT (0–32); Model 3 for NRT (0–32); Model 4 for NT-Post (0–20); Model 5 for NRT-Post (0–20)

According to the stratified analysis by gender and age group, the impact of NT in elders aged greater than 75 years (partial r = 0.369, *p* < 0.001) was highest among all DHI, follows by the impact of NT-Post in elders aged greater than 75 years (partial r = 0.359, *p* < 0.001) (Table 4). The impact of NT was lowered in males (partial r = 0.297, *p* < 0.001) and younger elders (partial r = 0.298, *p* < 0.001). The impact of NT-Post was lowered in males (partial r = 0.290, *p* < 0.001) and younger elders (partial r = 0.310, *p* < 0.001).

**Table 4:** Gender and age stratified association impact of dental health indicators on total occlusal force (n = 551)

Stratum variable (range)	β	SE	Partial r	*p* value	R^2^
Male (n = 165)					
Dental status (0–2)	− 72.91	40.87	− 0.14	0.08	0.14
NT (0–32)	13.65	3.50	0.30	**< 0.001**	0.20
NRT (0–32)	12.94	5.84	0.17	**0.03**	0.15
NT-Post (0–20)	20.49	5.40	0.29	**< 0.001**	0.20
NRT-Post (0–20)	15.94	7.59	0.17	**0.04**	0.15
Female (n = 386)					
Dental status (0–2)	− 130.22	27.58	− 0.24	**< 0.001**	0.09
NT (0–32)	16.15	2.33	0.34	**< 0.001**	0.14
NRT (0–32)	15.76	4.67	0.17	**0.001**	0.06
NT-Post (0–20)	23.90	3.40	0.34	**< 0.001**	0.15
NRT-Post (0–20)	19.24	5.47	0.18	**< 0.001**	0.06
Age < 75 (n = 226)					
Dental status (0–2)	− 138.40	44.21	− 0.21	**0.002**	0.11
NT (0–32)	15.33	3.33	0.30	**< 0.001**	0.15
NRT (0–32)	19.83	6.21	0.21	**0.002**	0.11
NT-Post (0–20)	23.51	4.88	0.31	**< 0.001**	0.16
NRT-Post (0–20)	26.03	7.68	0.22	**< 0.001**	0.12
Age > 75 (n = 325)					
Dental status (0–2)	− 110.19	25.09	− 0.24	**< 0.001**	0.10
NT (0–32)	16.15	2.28	0.37	**< 0.001**	0.18
NRT (0–32)	12.04	4.41	0.15	**0.006**	0.07
NT-Post (0–20)	23.45	3.42	0.36	**< 0.001**	0.17
NRT-Post (0–20)	13.98	5.32	0.15	**0.009**	0.07

Bold denotes statistically significant

*NT* number of natural teeth, *NTR* number of natural and rehabilitated teeth, *NT-Post* number of natural posterior teeth, *NRT-Post* number of natural and rehabilitated posterior teeth, ***β*** regression coefficient, *SE* standard error for **β**

Partial r obtained from linear regression model for occlusal force adjusted for age, gender, smoking, alcohol, education, periodontitis and metabolic syndrome

Dental Status: 0 = Dentate; 1 = Partial denture; 2 = Complete denture

The impact of dental status was higher in females (partial r = − 0.236, *p* < 0.001) and in older elders (partial r = − 0.240, *p* < 0.001), but lower in males (partial r = − 0.141, *p* = 0.076) and younger elders (partial r = − 0.207, *p* = 0.002).

The impact of NRT was higher in younger elders (partial r = 0.211, *p* = 0.002), but lower in older elders (partial r = 0.153, *p* = 0.006). The impact of NRT-Post was lower in males (partial r = 0.165, *p* = 0.038), but higher in females (partial r = 0.178, *p* < 0.001).

## Discussion

This study showed that TOF was 464.24 N for dentate, 297.15 N for PD, and 280.42 N for CD, which was associated with NT-Post and NT. According to this study, keeping NT, especially NT-Post, was the most critical factor for keeping TOF. This study indicated that 12 NT-Post are required for keeping the occlusal force for dentate. Moreover, gender and age group were effect modifiers of the association between DHI and TOF. Thus, TOF could be a robust indicator for oral health status. Also, this study supported the previous studies that TOF had a high relationship with the number of residual teeth [16, 28]. Moreover, this study is the first evidence to show the distribution and critically associated impact of DHI on objective TOF among Korean elders.

Objective TOF measurement was applied as compared to the previous methods of subjective occlusal force measurements because objective TOF measurement was a more reliable and valid approach of TOF measurement than subjective measurement [9]. Before pressure-sensitive films were developed, chewing cycle and chewing time were objective measurement methods of TOF [10]. Since chewing cycle and chewing time were time-consuming and difficult to use, subjective measurement of TOF was commonly applied.

The major strengths of this study were four-fold. Firstly, the number of participants was adequate to generalize the findings of this study to Korean elders. Secondly, participants were from a community-based health cohort, which could reduce the selection bias of the hospital-based participants. The odds of the disease have been higher for the hospital-based participants compared to the community-based participants. Thirdly, the examinations were performed by trained physicians and dentists, which showed high reliability for consistent measurement. Finally, the association was controlled for various confounders such as important socio-demographic factors such as age, gender and education, behavioral factors such as alcohol drinking, smoking, and health factors such as periodontitis and metabolic syndrome, which guaranteed the internal validity of this study.

When tooth loss occurs, rehabilitation of the lost tooth could ameliorate the decrease in TOF, which could not reach the original occlusal force before tooth loss. Increased occlusal force through rehabilitation of lost tooth could be speculated to reduce cognitive impairment risk [16]. Previous evidence [29, 30] showed that rehabilitation in denture increased occlusal force. Rehabilitative treatment with dental prosthesis such as denture is one of the viable treatment option as it is known for its functional support and increase in occlusal force [30]. Since this study was cross-sectional, this study could not rectify the previous studies. Hence, further pre-and post- rehabilitation clinical trials for the changes of occlusal force after rehabilitation of lost tooth should be indicated to prove the speculation.

To date, objective measurement for occlusal force and its effect on masticatory function was lacking for geriatric dentistry. Most of the studies involved a subjective assessment of the participant’s masticatory performance and efficiency [9, 28]. Although some studies used the Prescale system [11, 12, 31], they did not take confounders into consideration. Prescale system is indeed an objective method for producing occlusal force with high reliability, but it includes the procedure of cleansing artifacts in the film by examiners. This study showed that systematic training for evaluating the film could yield appropriate reliability (ICC = 0.96–0.97) on occlusal force measurement, which was similar to the reproducibility of TOF in previous studies [32–34]. Since the occlusal force measurement has clinical diagnostic value, it could measure the functional recovery objectively in cases of vertical dimensional loss for prosthodontic, orthodontic or orthognathic surgery [35]. In addition, TOF could be a useful tool to evaluate overall oral function in clinics and community screenings.

Gender and age group were effect modifiers of the association between DHI and OF. This study showed similar trends for males and females on TOF as previous results for Japanese elders [13, 36] TOF was higher in males than in female (427 N of TOF for males versus 400 N for females), which was comparable to Japanese evidence: 408 N for males and 244 N for females in 65–75 years Japanese elders [13], 502 N for males and 372 N females in 60–87 years Japanese elders [16]. Gender difference in TOF could be due to the females’ tendency to have weaker muscles of mastication compared to males. This study showed an age difference in TOF that younger elders had higher TOF than older elders. A Japanese study showed that age, rather than the number of remaining teeth, accounts for the lower occlusal force [28]. The influence of age on TOF has not been clarified yet. One study showed that there was no or little difference in terms of occlusal force and age group [14]. Another study showed that occlusal force increased until age 20, stabilized up to 40–50 years, then reduced due to the aging process, but there was no difference over 80 years for elders with 20 or more teeth [15]. This study supported the study that TOF was reduced with the increase of age in elders.

This study had some limitations. Firstly, TOF was measured with dentures in the mouth, so NT was a component of NRT. The TOF of real NT can be measured without dentures in the mouth, which can be indicated in further studies. Secondly, TOF could be affected by dynamic oral soft tissues, physical muscle, grip strength, and frailty of the elders [37], which could increase under-adjustment bias in the results. Occlusal force for elders was associated with walking speed [16] and functional ability [29]. Further studies including these factors could reduce this type of bias. Thirdly, we have not considered partial dentures having different types of support such as tissue-support, tooth-support or a combination of both according to Kennedy’s classification. Also, the quality of the dentures was not assessed meticulously. Although we envisaged that masticatory force could be very different across natural-natural, natural-artificial, and artificial-artificial tooth-pairs, this has not been into consideration. Finally, Prescale system is indeed an objective method for producing occlusal force with high reliability, but it includes erasing the artifacts in the film by examiners. The determination of artifacts is subjective and as such could be a limitation of the study. Despite the limitations, the data and analyses were sufficient to fulfil the aims of this study.

## Conclusions

Objective total occlusal force was associated with dental health indicators such as dental status, natural teeth, natural and rehabilitated teeth. Gender and age group modified its association. Total occlusal force could be an appropriate indicator for assessing overall dental health status.

## Data Availability

The datasets used and/or analyzed during the current study are available from the corresponding author on reasonable request.

## Abbreviations

DHI: Dental health indicator
DS: Dental status
NT: Natural teeth
NT-post: Natural posterior teeth
NRT: Natural and rehabilitated teeth
NRT-post: Natural and rehabilitated posterior teeth
TOF: Total occlusal force
PD: Partial denture
CD: Complete denture
CAL: Clinical attachment loss
ICC: Intra/inter-examiner reliability
SD: Standard deviation
r: Correlation coefficient
